# Comparative effectiveness of moderate-intensity statin with ezetimibe therapy versus high-intensity statin monotherapy in patients with acute coronary syndrome: a nationwide cohort study

**DOI:** 10.1038/s41598-024-51310-5

**Published:** 2024-01-08

**Authors:** Ji-Yong Jang, Seonji Kim, Jaehyeong Cho, Sung-youn Chun, Seng Chan You, Jung-Sun Kim

**Affiliations:** 1 Division of Cardiology, National Health Insurance Service Ilsan Hospital, Goyang, Korea; 2 Department of Biomedical Systems Informatics, Yonsei University College of Medicine, Seoul, Korea; 3 Institute for Innovation in Digital Healthcare, Yonsei University, Seoul, Korea; 4 Department of Research and Analysis, National Health Insurance Service Ilsan Hospital, Goyang, Korea; 5 Division of Cardiology, Severance Hospital, Yonsei University College of Medicine, Seoul, Korea

**Keywords:** Cardiology, Diseases, Health care, Medical research

## Abstract

The long-term outcome of first-line moderate-intensity statin with ezetimibe combination therapy for secondary prevention after percutaneous coronary intervention in patients with acute coronary syndrome (ACS) compared to high-intensity statin monotherapy remains elusive. The objective of this study was to compare the effectiveness of moderate-intensity statin and ezetimibe combination therapy with high-intensity statin monotherapy. We conducted a nationwide, population-based, retrospective, cohort study of patients with ACS from 2013 to 2019. The patients using combination therapy were matched (1:1) to those using monotherapy. The primary outcome was a composite of myocardial infarction, stroke and all-cause mortality. We estimated the hazard ratios (HR) and 95% confidence intervals (CIs) using the Cox proportional hazards regression. After propensity score matching, 10,723 pairs were selected. Men accounted for 70% of the patients and 37% aged > 70 years. The primary endpoint occurred in 1297 patients (12.1%) in the combination group and in 1426 patients (13.3%) in the monotherapy group, and decreased risk (HR 0.85, 95% CI 0.78–0.92, *P* < 0.001) in the combination group. Among the patients with ACS, moderate-intensity statin with ezetimibe combination therapy was associated with decreased risk of adverse cardiovascular outcomes compared with high-intensity statin monotherapy in a nationwide population-based study representing routine clinical practice.

## Introduction

The idea of ‘lower is better’ has become a central dogma in secondary prevention of coronary atherosclerotic disease^1,2^. Reducing low-density lipoprotein cholesterol by using high-dose 3-hydroxy-3-methylglutaryl-coenzyme A reductase inhibitors, is one of the cornerstones in patient management after percutaneous coronary intervention^3,4^. Decreased low-density lipoprotein cholesterol levels with high-intensity statin therapy has been proven to prevent secondary atherosclerotic cardiovascular disease in a dose–response manner^5,6^. However, the evidence has been poorly translated into clinical practice. Many clinicians and patients alike are reluctant to initiate and maintain high-intensity statins for secondary prevention because of anxiety to a higher rate of adverse drug related events and psychological burden to high-intensity, even after percutaneous coronary intervention, and often require additional treatment that includes ezetimibe^7,8^.

After the IMPROVE-IT (Improved Reduction of Outcomes: Vytorin Efficacy International Trial) demonstrated the superior efficacy of combined moderate-intensity statin and ezetimibe therapy compared with moderate-intensity statin monotherapy in hospitalized patients with acute coronary syndrome^9^, current guidelines recommend that in cases where the optimal low-density lipoprotein goal is not achieved, ezetimibe therapy should be added to the maximally tolerated statin dose^10^. However, this trial was conducted to assess the comparison between moderate-intensity statin monotherapy and combination therapy, therefore the effectiveness of moderate-intensity statin with ezetimibe combination therapy compared to of high-intensity statin monotherapy remains unclear.

The recent the randomized comparison of efficacy and safety of lipid lowering with statin monotherapy versus statin–ezetimibe combination for high-risk cardiovascular disease (RACING) trial demonstrated the comparable long-term efficacy and safety of high-intensity statin monotherapy versus moderate-intensity statin with ezetimibe combination therapy in patients with stable status of atherosclerotic cardiovascular disease^11^. However, patient compliance with the prescribed lipid-lowering therapy and the proportion of patients with low-density lipoprotein cholesterol concentration levels < 70 mg/dL was higher in the moderate-intensity statin with ezetimibe combination therapy group than in the high-intensity statin monotherapy group^11^. Nonetheless, there is still uncertainty about safety for using moderate-intensity statin with ezetimibe combination therapy instead of high-intensity monotherapy focused on the patients with acute coronary syndrome even in immediately after percutaneous coronary intervention.

This study aimed to compare the effectiveness to reduction of cardiovascular outcome and drug adherence of moderate-intensity statin with ezetimibe combination therapy with high-intensity statin monotherapy in patients who underwent percutaneous coronary intervention with acute coronary syndrome in routine clinical practice.

## Materials and methods

### Data sources

This study was a retrospective analysis based on the Korean National Health Claims database established by the National Health Insurance Service (NHIS). The NHIS, a single insurer run by the Korean government, includes 97.1% of Korean citizens, with the remaining 3% designated as those in need of medical assistance. This database includes comprehensive data on patient sociodemographic information, inpatient and outpatient services, drug prescriptions, procedures, and mortality^12^. Diagnostic variables are recoded according to the International Classification of Diseases, 10th revision (ICD‐10) codes, and data with anonymized identifiers were provided to the researchers.

### Cohort design and study population

**Figure 1:**
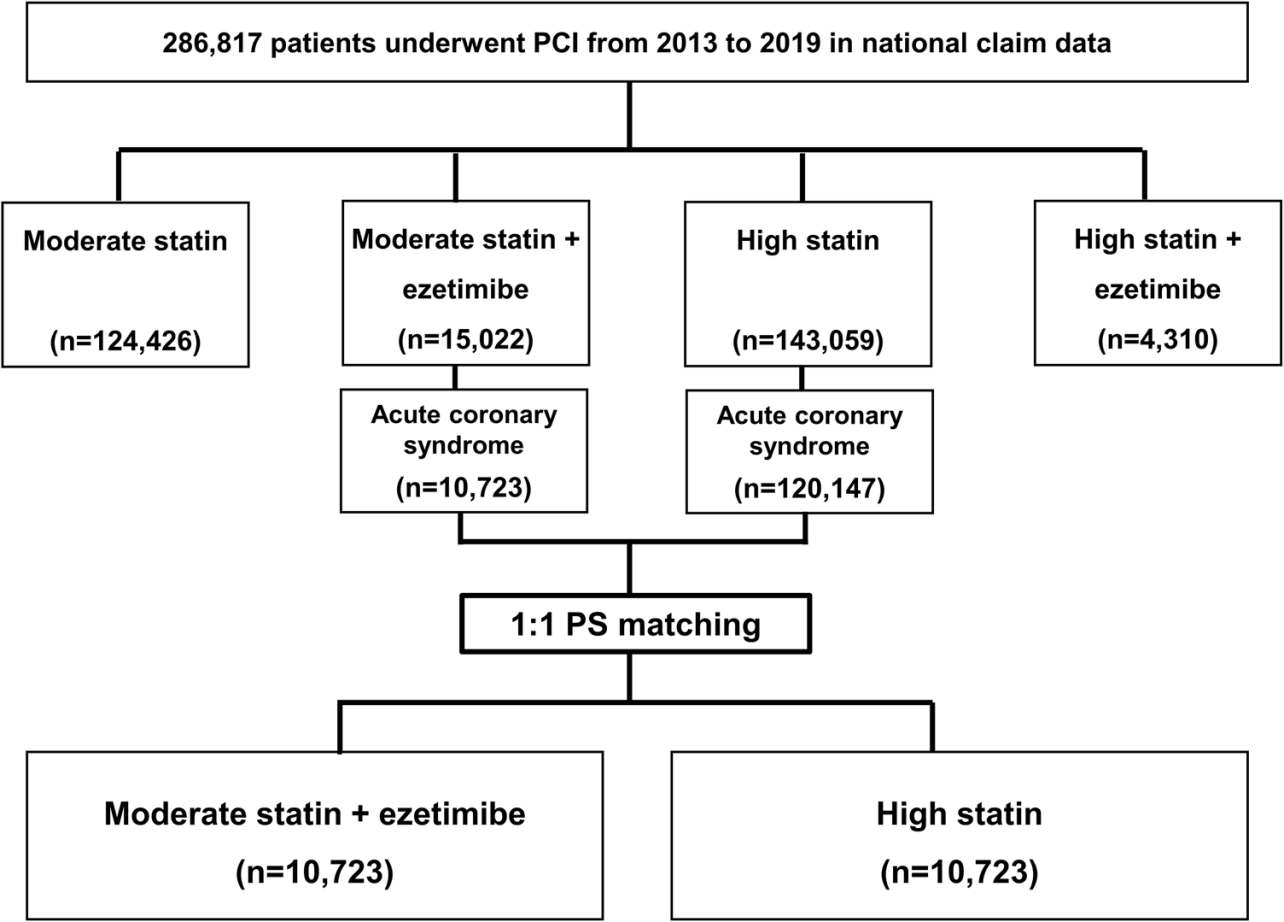
Flow chart of the study population. PCI, percutaneous coronary intervention; PSM, propensity score matching.

We conducted a nationwide, population-based, retrospective cohort study. We enrolled patients who underwent percutaneous coronary intervention with a diagnosis of acute coronary syndrome between January 1, 2013, and December 31, 2019 (Fig. 1). The lipid-lowering strategy was characterized at hospital discharge (based on the most frequently prescribed drug group during hospitalization) as follows: moderate-intensity statin monotherapy, moderate-intensity statin with ezetimibe combination therapy, high-intensity statin monotherapy, and high-intensity statin with ezetimibe combination therapy (Supplementary Table S1). The statin intensity was prescribed according to the 2013 American College of Cardiology and American Heart Association guidelines^13^. The moderate-intensity statin with ezetimibe combination therapy and high-intensity statin monotherapy groups were restricted to acute coronary syndrome and matched using 1:1 propensity score matching.

### Outcome and covariates

The primary outcome was a composite of myocardial infarction, ischemic stroke, and all-cause mortality (Supplementary Table S2)^11^. Secondary outcomes included individual outcomes of the primary outcome.

Deaths during hospitalization for percutaneous coronary intervention were excluded from the analysis. Myocardial infarction was defined as a case in which the cardiovascular procedure code and a new diagnosis of myocardial infarction as the main disease were recorded. Stroke was defined as both new hospitalization for and diagnosis of stroke as the main disease. Event definitions and covariates are presented in Supplementary Table S2.

Patient history was extracted from the date of percutaneous coronary intervention and backtracked up to 2 years prior to intervention. A clinical presentation was considered as newly diagnosed if the diagnosis was made within 1 week before the percutaneous coronary intervention hospitalization period. If there were two or more diagnoses, the more severe diagnosis was selected. Drug adherence was defined as patients who collected at least 80% of the prescribed statin monotherapy or combination therapy for 3 months after discharge for percutaneous coronary intervention.

### Statistical analysis

The frequency and proportion of patients were reported according to baseline characteristics, comorbidity, revascularization, clinical presentation at index procedure, year of percutaneous coronary intervention, and drug adherence, which equal or exceeded 80% among the study and matched populations. Categorical variables were performed using the chi-square test and presented as means and standard deviations and were analyzed using the independent t-test and analysis of variance test. Propensity scores were calculated by logistic regression for baseline characteristics, the size of the hospital, potent P2Y12 inhibitor use and years of percutaneous coronary intervention where the patients received treatment. We considered 1:1 propensity score matching as the nearest neighbor with calipers of width of 0.2 standard deviations of the logit of the propensity score between the moderate-intensity statin with ezetimibe and high-intensity statin groups. A calculated standardized difference of < 0.1 was considered acceptable.

Survival analysis was conducted using the Kaplan–Meier method with log-rank test. The follow-up period was defined from the index date until the occurrence of the outcome, or the last date of the study (December 31, 2020), whichever came first. The proportional hazards assumption was tested using the by log− log survival plots, and the violations of the assumption were not found for the outcome. The hazard ratios (HR) and 95% confidence intervals (CIs) were assessed using the Cox proportional hazards regression model among the matched populations. We adjusted for drug adherence for statin monotherapy or combination therapy (≥ 80%) to control for confounders. Statistical significance was defined as a two-sided *p*-value < 0.05.

In the subgroup analysis, the risk according to age (≥ 70 years), sex, myocardial infarction history, diabetes mellitus, acute myocardial infarction, and drug adherence was calculated, and the interaction with statin therapy was analyzed using the *p*-value for interaction. As a sensitivity analysis, we used an inverse probability of treatment weighting approach based on baseline characteristics and the size of the hospital to calculate the risk of adverse outcomes on the weighted population. Database construction and statistical analyses were performed using SAS version 9.3 (SAS Institute, Cary, NC, USA). The study was performed in accordance with the Declaration of Helsinki and reported in compliance with the Strengthening the Reporting of Observational Studies in Epidemiology (STROBE) guidelines.

### Ethical approval

This study was approved and the requirement for informed consent was waived because of the retrospective nature of the study by the Institutional Review Board of the National Health Insurance Service Ilsan Hospital (IRB No. 2021-03-024).

## Results

### Patient characteristics

We identified 286,817 patients who underwent percutaneous coronary intervention between 2013 and 2019; 124,426 patients (43.4%) received moderate-intensity statin, 15,022 patients (5.2%) received combined moderate-intensity statin with ezetimibe, 143,059 patients (49.9%) received high-intensity statin, and 4310 patients (1.5%) received combined high-intensity statin with ezetimibe (Fig. 1, Table 1). The combined use of moderate-intensity statins with ezetimibe has steadily increased in clinical practice. The 60–69-year-old group was the largest (28.7%), and 71.2% of the patients were male.

**Table 1:** Baseline characteristics of patients with acute coronary syndrome who underwent percutaneous coronary intervention.

Statin group	Moderate statin	Moderate statin + ezetimibe	High statin	High statin + ezetimibe
Participants	124,426 (100.0)	15,022 (100.0)	143,059 (100.0)	4310 (100.0)
Male sex	84,940 (68.3)	10,580 (70.4)	105,590 (73.8)	3228 (74.9)
Age groups				
≤ 40 s	10,420 (8.4)	1319 (8.8)	17,417 (12.2)	570 (13.2)
50 s	26,287 (21.1)	3424 (22.8)	35,113 (24.5)	1165 (27.0)
60 s	35,991 (28.9)	4831 (32.1)	40,130 (28.1)	1248 (29.0)
70 s	35,671 (28.7)	3863 (25.7)	34,493 (24.1)	894 (20.7)
≥ 80 s	16,057 (12.9)	1585 (10.6)	15,906 (11.1)	433 (10.1)
Comorbidity and revascularization				
Diabetes mellitus	46,553 (37.4)	5658 (37.7)	51,026 (35.7)	1654 (38.4)
Hypertension	106,925 (85.9)	12,989 (86.5)	119,945 (83.8)	3441 (79.8)
MI	33,956 (27.3)	4166 (27.7)	67,637 (47.3)	1792 (41.6)
Heart failure	30,431 (24.5)	3082 (20.5)	32,048 (22.4)	1040 (24.1)
PAD	9695 (7.8)	1302 (8.7)	8801 (6.2)	295 (6.8)
ESRD	4548 (3.7)	369 (2.5)	3747 (2.6)	100 (2.3)
Prior PCI	9830 (7.9)	672 (4.5)	12,252 (8.6)	454 (10.5)
Prior CABG	10,053 (8.1)	691 (4.6)	12,401 (8.7)	456 (10.6)
Clinical presentation at index procedure				
STEMI	25,820 (20.8)	3124 (20.8)	36,251 (25.3)	1062 (24.6)
NSTEMI	25,921 (20.8)	3236 (21.5)	47,975 (33.5)	1294 (30.0)
Unstable angina	31,894 (25.6)	3750 (25.0)	26,452 (18.5)	881 (20.4)
Stable angina	29,739 (23.9)	3676 (24.5)	20,150 (14.1)	748 (17.4)
Others	11,052 (8.9)	1236 (8.2)	12,231 (8.5)	325 (7.5)
Potent P2Y12 inhibitor	9515 (7.7)	1386 (9.2)	24,555 (17.2)	666 (15.5)
Years of PCI				
2013	20,137 (16.2)	843 (5.6)	12,587 (8.8)	0 (0.0)
2014	19,796 (15.9)	739 (4.9)	17,341 (12.1)	0 (0.0)
2015	17,898 (14.4)	791 (5.3)	19,183 (13.4)	0 (0.0)
2016	18,658 (15.0)	1586 (10.6)	21,190 (14.8)	206 (4.8)
2017	17,009 (13.6)	3173 (21.1)	22,601 (15.8)	768 (17.8)
2018	15,886 (12.8)	3518 (23.4)	24,504 (17.1)	1248 (29.0)
2019	15,042 (12.1)	4372 (29.1)	25,653 (18.0)	2088 (48.4)

Data are number (%).

*MI* myocardial infarction, *PAD* peripheral artery disease, *ESRD* end-stage renal disease, *PCI* percutaneous coronary intervention, *CABG* coronary artery bypass graft, *STEMI* ST-elevation myocardial infarction, *NSTEMI* non-ST elevation myocardial infarction.

Patients in the high-intensity statin group were more likely to have myocardial infarction (47.3% vs. 27.7%), heart failure (22.4% vs. 20.5%), and revascularization (8.6% vs. 4.5% with percutaneous coronary intervention and 8.7% vs. 4.6% with coronary artery bypass graft) than the moderate-intensity statin with ezetimibe group (Table 1). Patients in the high-intensity statin group had higher prevalence of ST-elevation myocardial infarction (27.7% vs. 22.7%), non-ST-elevation myocardial infarction (36.7% vs. 23.4%), and potent P2Y12 inhibitor use (17.2% vs. 9.2%) than the moderate-intensity statin with ezetimibe group.

Using 1:1 propensity score matching, 10,723 pairs were selected among patients who were prescribed monotherapy high-intensity statin and combined moderate-intensity statin with ezetimibe therapy after percutaneous coronary intervention for acute coronary syndrome (Fig. 1, Table 2). After propensity score matching, maximum standardized mean difference was 0.07 (Table 2). The propensity score distribution is shown in Supplementary Fig. S1. The mean follow-up duration was 1028 days in the moderate-intensity statin with ezetimibe group and 1026 days in the high-intensity statin group (Table 2). Approximately 70% of the patients were male, more than one-third were ≥ 70 years of age, 12% were receiving potent P2Y12 inhibitors, and 58% were treated at tertiary hospitals. Of the clinical presentations at the index procedure, unstable angina was the most common, followed by non-ST-elevation myocardial infarction, and ST-elevation myocardial infarction. The proportion of patients undergoing percutaneous coronary intervention between 2013 and 2019 increased from 6.0 to 28.2% in the moderate-intensity statin with ezetimibe group and from 5.8 to 28.1% in the high-intensity statin group.

**Table 2:** Baseline characteristics of the matched population.

Statin group	Moderate Statin + Ezetimibe	High Statin	Standardized Difference
Participants	10,723 (100.0)	10,723 (100.0)	
Follow-up duration, days	1028 ± 654	1026 ± 645	
Male sex	7544 (70.4)	7590 (70.8)	− 0.01
Age groups			0.00
≤ 40 s	1015 (9.5)	994 (9.3)	
50 s	2489 (23.2)	2459 (22.9)	
60 s	3307 (30.8)	3311 (30.9)	
70 s	2714 (25.3)	2734 (25.5)	
≥ 80 s	1198 (11.2)	1225 (11.4)	
Comorbidity and revascularization			
Diabetes mellitus	3546 (33.1)	3516 (32.8)	0.01
Hypertension	9245 (86.2)	9305 (86.8)	− 0.02
Old MI	3967 (37.0)	3962 (37.0)	0.00
Heart failure	1788 (16.7)	1737 (16.2)	0.01
PAD	891 (8.3)	829 (7.7)	0.02
ESRD	270 (2.5)	167 (1.6)	0.07
Prior PCI	507 (4.7)	459 (4.3)	0.02
Prior CABG	521 (4.9)	462 (4.3)	0.03
Clinical presentation at index procedure			
STEMI	1828 (17.0)	1851 (17.3)	− 0.01
NSTEMI	3076 (28.7)	3089 (28.8)	0.00
Unstable angina	4563 (42.6)	4538 (42.3)	0.00
Others	1256 (11.7)	1245 (11.6)	0.00
Potent P2Y12 inhibitor	1255 (11.7)	1245 (11.6)	0.03
Tertiary general hospital	6247 (58.3)	6189 (57.7)	0.01
Years of PCI			0.05
2013	645 (6.0)	625 (5.8)	
2014	562 (5.3)	573 (5.3)	
2015	578 (5.4)	593 (5.5)	
2016	1199 (11.2)	1231 (11.5)	
2017	2245 (20.9)	2256 (21.0)	
2018	2469 (23.0)	2439 (22.8)	
2019	3025 (28.2)	3006 (28.1)	

Data are number (%) or mean ± standard deviation (SD).

MI, myocardial infarction; PAD, peripheral artery disease; ESRD, end-stage renal disease; PCI, percutaneous coronary intervention; CABG, coronary artery bypass graft; STEMI, ST-elevation myocardial infarction; NSTEMI, non-ST elevation myocardial infarction.

### Comparative effectiveness

**Figure 2:**
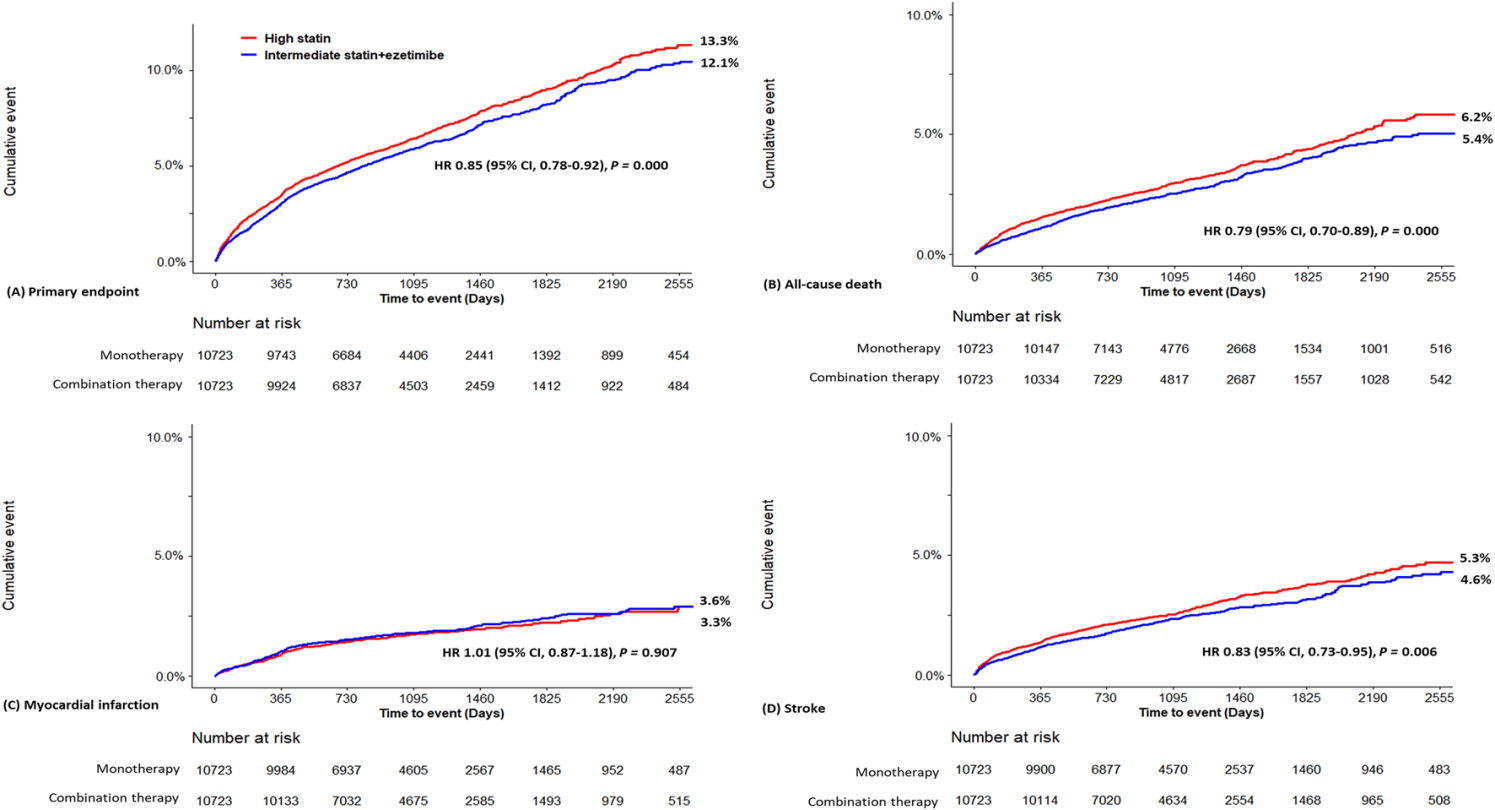
Kaplan–Meier curves of the primary endpoint in matched population.

The cumulative incidence curves for the major adverse cardiovascular event are shown in Fig. 2. Among the matched patients, the primary endpoints, including myocardial infarction, ischemic stroke, and all-cause mortality, occurred in 1,297 patients (4.29 events per 100 person-year) in the moderate-intensity statin with ezetimibe combination group and in 1426 patients (4.73 events per 100 person-year) in the high-intensity statin group (Table 3). The risk and incidence of the primary outcome were significantly lower (HR 0.85, 95% CI 0.78–0.92) in the moderate-intensity statin with ezetimibe combination group. The risk of all-cause mortality (HR 0.79, 95% CI 0.70–0.89) and stroke (HR 0.83, 95% CI 0.73–0.95) were lower in the combination group. There was no difference in the risk of myocardial infarction (HR 1.01, 95% CI 0.87–1.18) between the two groups.

**Table 3:** Risk of clinical outcomes and description of statin adherence in the matched population.

	Moderate Statin + Ezetimibe (n = 10,723)	High Statin (n = 10,723)	Hazard ratio	*P* value
Primary endpoint—Composite of all-cause death, myocardial infarction, and stroke	1297 (12.1)	1426 (13.3)	0.85 (0.78–0.92)	0.000
All-cause death	574 (5.4)	664 (6.2)	0.79 (0.70–0.89)	0.000
Myocardial infarction	382 (3.6)	358 (3.3)	1.01 (0.87–1.18)	0.907
Stroke	493 (4.6)	564 (5.3)	0.83 (0.73–0.95)	0.006
Drug adherence (≥ 80%)	7,276 (67.8)	5,363 (50.0)		0.000

Data are number (%).

Hazard ratio for moderate statin + ezetimibe group was derived from Cox proportional analysis.

Drug adherence was defined as patients who collected at least 80% of the prescribed statin monotherapy or combination therapy for 3 months after hospitalization for percutaneous coronary intervention.

Based on the 90-day statin prescription, statin compliance in the moderate-intensity statin with ezetimibe combination therapy group was higher than that in the high-intensity statin monotherapy group (67.8% vs. 50.0%, *p* < 0.001).

### Subgroup analyses

**Figure 3:**
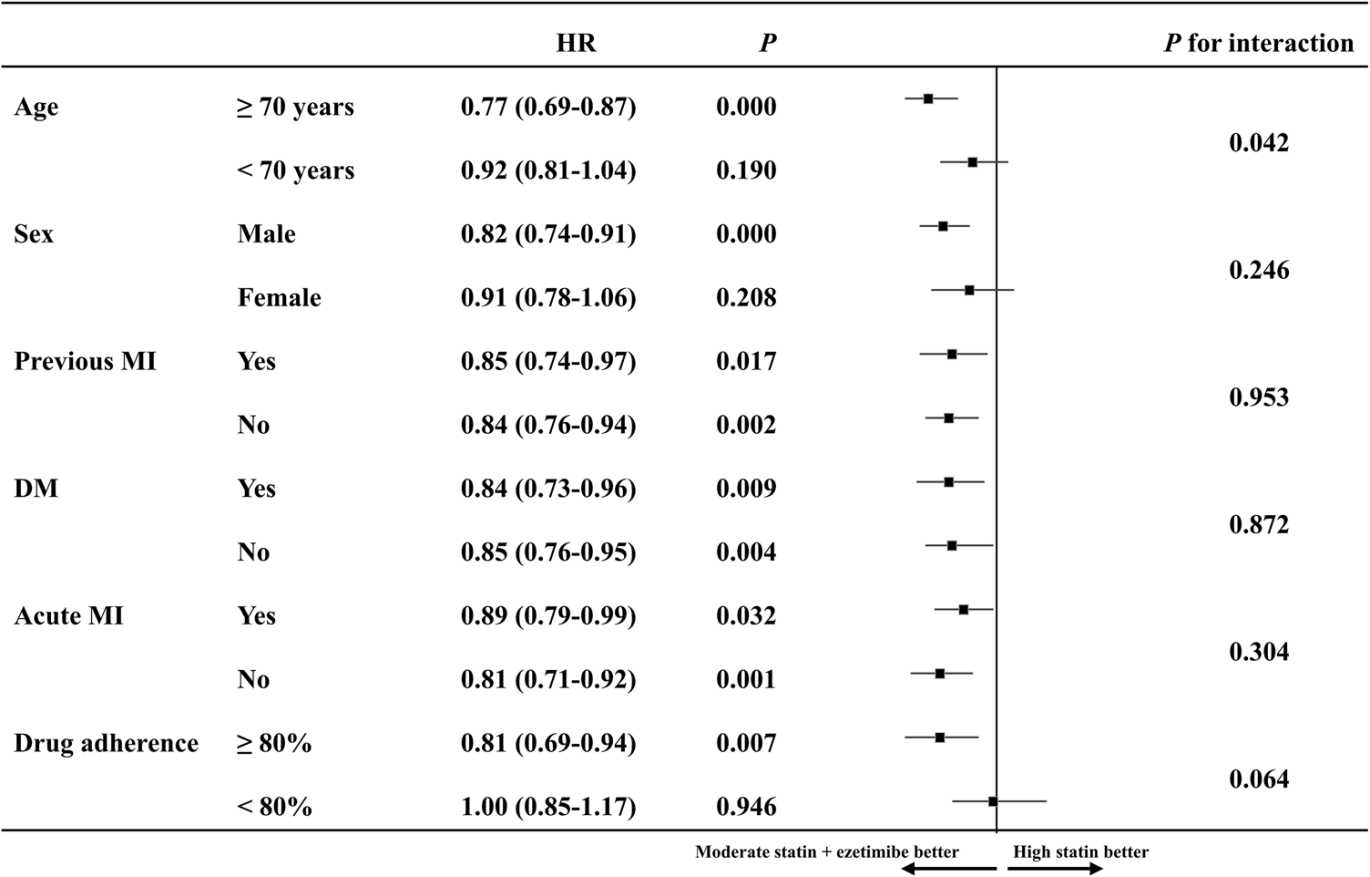
Subgroups analysis for the primary endpoint in matched population. HR, Hazard ratio; MI, myocardial infarction; DM, diabetes mellitus.

A subgroup analysis was performed according to the primary endpoint of the matched population by age (≥ 70 years), sex, myocardial infarction history, diabetes mellitus, acute myocardial infarction, and drug adherence (Fig. 3). The risk of the primary endpoint showed consistent result by myocardial infarction history, diabetes mellitus and acute myocardial infarction. There was no interaction effect in any subgroup.

### Sensitivity analyses

The population also standardized differences between the matched groups were balanced for identified confounders, except for hospital size, after the inverse probability of treatment weighting (Supplementary Table S3). The risks of primary endpoint (HR 0.91, 95% CI 0.86–0.95) and all-cause mortality (HR 0.86, 95% CI 0.80–0.93) were lower in the combination group, and the results showed similar pattern with the propensity score matching (Supplementary Table S4). In the subgroup analysis, risk of the primary endpoint was decreased in the all subgroups excepts to female (HR 0.98, 95% CI 0.90–1.07) and low drug adherence (HR 0.97, 95% CI 0.90–1.04).

## Discussion

In this study, we found that (i) the risks of major adverse cardiovascular event were decreased in the use of moderate-intensity statin with ezetimibe combination therapy compared to high-intensity statin monotherapy in patients who underwent percutaneous coronary intervention for acute coronary syndrome. The risk in all-cause mortality and stroke were also lower; (ii) the moderate-intensity statin with ezetimibe combination therapy group showed a higher rate of drug compliance for 90 days after discharge; (iii) regardless of myocardial infarction history, diabetes mellitus and acute myocardial infarction, the risks of cardiovascular composite outcomes decreased in the ezetimibe combination therapy, and there was no interaction between each subgroup and treatment strategies. To the best of our knowledge, this study enrolled the largest population representing nationwide clinical practice.

The results of this study are comparable to those of previous interventional and observational studies. A Chinese randomized clinical trial found superior efficacy of moderate-intensity statin with ezetimibe combination therapy over high-intensity statin monotherapy in decreasing low-density lipoprotein cholesterol levels in stable patients^14^. Observational studies, which enrolled patients with combination lipid-lowering therapy and included < 1000 patients, also found comparable effectiveness of the two therapy options after acute myocardial infarction or percutaneous coronary intervention^15–17^. The recent RACING trial demonstrated the non-inferiority of combination therapy compared with high-intensity statin in patients with stable atherosclerotic cardiovascular disease with a higher control rate of low-density lipoprotein cholesterol level and therapy adherence in the combination therapy group^11^. In this clinical trial, included subjects with various conditions for vascular disease which was not specified to patients underwent percutaneous coronary intervention. Therefore, although numerous studies have explored high-intensity monotherapy and moderate-intensity combination treatment to reduce lipid levels for secondary prevention, there remains insufficient evidence for the preemptive use of combination therapy in high-risk patients who have undergone stent implantation for acute coronary syndrome^9,18^. In this regard, we established evidence for the additional benefits of combining moderate-intensity statins with ezetimibe in acute coronary syndrome patients.

Current guidelines recommend initiating and maintaining high-potency statins, which can decrease low-density lipoprotein cholesterol levels by > 50%. As the benefit of statin therapy largely relies on individual absolute risks, it is important to reinforce prolonged statin treatment in high-risk patients, such as patients undergoing percutaneous coronary intervention^5^. Nonetheless, the progress toward improved lipid management in routine clinical practice has been slower than expected. Among Medicare beneficiaries hospitalized for myocardial infarction, less than two-thirds adhered to high-intensity statin therapy at 6 months (58.9%) and 2 years (41.6%) after discharge^7^.

Because of the high failure rate in achieving the low-density lipoprotein target in patients with atherosclerotic cardiovascular disease, the 2019 European Society of Cardiology guidelines recommend adding ezetimibe therapy to the maximally tolerated intensity of statin. However, this study, which represents routine clinical practice in Korea and was performed before the publication of the RACING trial, identified an increasing trend of favoring moderate-intensity statin with ezetimibe combination therapy over high-intensity statin monotherapy in patients undergoing percutaneous coronary intervention. This trend of favoring combination therapy has been previously demonstrated in the treatment of hypertension.

Since 2018, the European Society of Cardiology/European Society of Hypertension guidelines recommend initiating antihypertensive drug treatment with combination therapy rather than sequential monotherapy or the stepped-care strategy^19^. Of note, this recommendation was based on weak supporting evidence from a small-sized randomized controlled trial^20^ and large observational studies^21,22^, which reported that initial combination therapy is better in achieving target blood pressure than sequential monotherapy or stepped-care strategies without clear demonstration of hard clinical endpoints. The reasons for favoring initial combination therapy over add-on therapy for hypertension may include the following: (1) the well-established relationship between blood pressure control and clinical endpoints and additional evidence supporting the idea that ‘lower is better’ in blood pressure; (2) accumulated evidence of the importance of treatment adherence; (3) the reluctance of physicians to prescribe high-intensity antihypertensive medication; (4) physician or treatment inertia can lead to suboptimal completion of the treatment regimen; (5) the presence of a single-pill combination^23^. This hypertension management approach can be applied to the management of hyperlipidemia.

This study had several limitations. Although propensity scoring matched the well-balanced baseline characteristics of the two groups, the possibility of unadjusted bias cannot be ruled out. And, the size of the two groups was different. Therefore, we tried to reduce the discrepancy between the two groups by conducting 1:1 propensity score matching and inverse probability of treatment weighting approach. Second, validation of diagnosis was not performed to confirm the outcome in the study population during follow-up due to the use of a de-identified claim database. However, the accuracy in definitions of stroke and recurrent acute myocardial infarction was reported in previous studies with similar outcome incidence, with more than 90.5% and 92.0% positive predictive value in the NHIS database^24,25^. Third, since no laboratory data are available, it is unknown whether the low-density lipoprotein-lowering effect of combination therapy was greater than that of the high-intensity statin group. Fourth, the results of this study do not guarantee generalizability of finding beyond Korea.

## Conclusions

In this observational study, moderate-intensity statin therapy with ezetimibe combination therapy provides a benefit over high-intensity statin monotherapy for the risk of adverse cardiovascular outcomes and all-cause mortality in the patients after percutaneous coronary intervention for acute coronary syndrome. These finding support a use of statin and ezetimibe combination therapy for patients with acute coronary syndrome.

### Supplementary Information

Supplementary Information.

## Data Availability

The data used in this study are third-party data owned and operated by the Korean NHIS. Interested researchers can contact the NHIS to access the data (http://nhiss.nhis.or.kr/bd/ab/bdabd003cv.do).

## Abbreviations

RACING trial: Randomized comparison of efficacy and safety of lipid lowering with statin monotherapy versus statin–ezetimibe combination for high-risk cardiovascular disease trial
IMPROVE-IT: Improved Reduction of Outcomes: Vytorin Efficacy International Trial
NHIS: National Health Insurance Service
HR: Hazard ratio
CI: Confidence interval
