# Supplementary material for: Comparative effectiveness of moderate-intensity statin with ezetimibe therapy versus high-intensity statin monotherapy in patients with acute coronary syndrome: a nationwide cohort study
Source: Sci Rep. 2024 Jan 8;14:838. doi: 10.1038/s41598-024-51310-5 (PMC10774297; doi:10.1038/s41598-024-51310-5)
Supplement: Supplementary file 1 — Supplementary Information. [file 41598_2024_51310_MOESM1_ESM.docx]

**Supplemental materials**

**Comparative effectiveness of moderate-intensity statin with ezetimibe therapy versus high-intensity statin monotherapy in patients with acute coronary syndrome: A nationwide cohort study**

Ji-Yong Jang^1†^, Seonji Kim^2,3†^, Jaehyeong Cho^2,3^, Sung-youn Chun^4^, Seng Chan You^2,3,*^, Jung-Sun Kim ^5,*^

^1^ Division of Cardiology, National Health Insurance Service Ilsan Hospital, Goyang, Korea

^2^ Department of Biomedical Systems Informatics, Yonsei University College of Medicine, Seoul, Korea

^3^ Institute for Innovation in Digital Healthcare, Yonsei University, Seoul, Korea

^4^ Department of Research and Analysis, National Health Insurance Service Ilsan Hospital, Goyang, Korea

^5^ Division of Cardiology, Severance Hospital, Yonsei University College of Medicine, Seoul, Korea

^†^ These authors contributed equally to this work.

**Supplementary Table S1.** Exposure drugs description

| **Variables** | **Dosage** |
| --- | --- |
| **Moderate-intensity statin monotherapy** | |
| Atorvastatin | 10mg |
| Atorvastatin | 20mg |
| Atorvastatin + amlodipine | 10mg + 5mg |
| Atorvastatin + S-amlodipine | 10mg + 2.5mg |
| Atorvastatin + amlodipine | 20mg + 5mg |
| Atorvastatin + amlodipine | 20mg + 10mg |
| Atorvastatin + irbesartan | 10mg + 0.15g |
| Atorvastatin + irbesartan | 10mg + 0.3g |
| Atorvastatin + irbesartan | 20mg + 0.15g |
| Atorvastatin + irbesartan | 20mg + 0.3g |
| Fluvastatin | 80mg |
| Pitavastatin | 2mg |
| Pitavastatin | 4mg |
| Pitavastatin + valsartan | 2mg + 80mg |
| Pitavastatin + valsartan | 2mg + 160mg |
| Pitavastatin + valsartan | 4mg + 80mg |
| Pitavastatin + valsartan | 4mg + 160mg |
| Pravastatin | 40mg |
| Pravastatin + fenofibrate | 40mg + 0.16g |
| Rosuvastatin | 5mg |
| Rosuvastatin | 10mg |
| Rosuvastatin + olmesartan | 5mg + 10mg |
| Rosuvastatin + olmesartan | 5mg + 20mg |
| Rosuvastatin + olmesartan | 10mg + 10mg |
| Rosuvastatin + olmesartan | 10mg + 20mg |
| Rosuvastatin + telmisartan | 5mg + 40mg |
| Rosuvastatin + telmisartan | 5mg + 80mg |
| Rosuvastatin + telmisartan | 10mg + 40mg |
| Rosuvastatin + telmisartan | 10mg + 80mg |
| Rosuvastatin + valsartan | 5mg + 80mg |
| Rosuvastatin + valsartan | 5mg + 160mg |
| Rosuvastatin + valsartan | 10mg + 80mg |
| Rosuvastatin + valsartan | 10mg + 160mg |
| Simvastatin | 20mg |
| Simvastatin | 40mg |
| Simvastatin | 80mg |
| Simvastatin + fenofibrate | 20mg + 0.145g |
| Simvastatin + fenofibrate | 40mg + 0.145g |
| **Moderate-intensity statin with ezetimibe combination therapy** | |
| Atorvastatin + ezetimibe | 10mg + 10mg |
| Atorvastatin + ezetimibe | 20mg + 10mg |
| Rosuvastatin + ezetimibe | 5mg + 10mg |
| Rosuvastatin + ezetimibe | 10mg + 10mg |
| Simvastatin + ezetimibe | 20mg + 10mg |
| Simvastatin + ezetimibe | 40mg + 10mg |
| **High-intensity statin monotherapy** | |
| Atorvastatin | 40mg |
| Atorvastatin | 80mg |
| Atorvastatin + amlodipine | 40mg + 5mg |
| Rosuvastatin | 20mg |
| Rosuvastatin + olmesartan | 20mg + 20mg |
| Rosuvastatin + olmesartan | 20mg + 40mg |
| Rosuvastatin + valsartan | 20mg + 80mg |
| Rosuvastatin + valsartan | 20mg + 160mg |
| Rosuvastatin + telmisartan | 20mg + 40mg |
| Rosuvastatin + telmisartan | 20mg + 80mg |
| **High-intensity statin with ezetimibe combination therapy** | |
| Atorvastatin + ezetimibe | 40mg + 10mg |
| Rosuvastatin + ezetimibe | 20mg + 10mg |

**Supplementary Table S2.** Covariates and patient outcomes

| **Variables** | **Code** | **Further description** |
| --- | --- | --- |
| **Comorbidity and revascularization (diagnosis during 2 years before PCI intervention)** | | |
| Diabetes mellitus | ICD-10: E10, E11, E12, E13, E14 | Patients with diabetes mellitus and prescribed antidiabetic drugs |
| Hypertension | ICD-10: I10, I11, I12, I13, I15 | Patients with hypertension and prescribed antihypertensive drugs |
| Old MI | ICD-10: I21, I22, I252 |  |
| Heart failure | ICD-10: I110, I130, I50, I971 |  |
| PAD | ICD-10: I700, I701, I702, I708, I709 |  |
| ESRD | EDI: O7011, O7012, O7013, O7014, O7015, O7016, O7017, O7018, O7020, O7021, O7061, O7062, O7074, AZ231, D0327002, E6593, IB510, IB511, IB520, IB530 |  |
| Prior PCI | ICD-10: Z955, Z958, Z959  and  EDI: M6551, M6552, M6561, M6563, M6562, M6564, M6553, M6554, M6565, M6566, M6567, M6571, M6572 |  |
| Prior CABG | ICD-10: T822, Z951, Z95  and  EDI: OA63, OB63, OA641, OA642, OA647, O016, O0170, O1641, O1642, O1643, O1644, O1645, O1646, O1647 |  |
| **Clinical presentation at index procedure** | | |
| STEMI | ICD-10: I21, I210, I211, I222, I223  and  EDI: M6553, M6565 |  |
| NSTEMI | ICD-10: I214, I219, I22, I220, I221, I228, I229, I252 |  |
| Unstable angina | ICD-10: I200 |  |
| Stable angina | ICD-10: I20, I201, I208, I209 |  |
| **Outcome** | | |
| Primary endpoint | Composite of all-cause death, myocardial infarction, or stroke |  |
| All-cause death | - | Patients were died from first PCI intervention date to last date of the study |
| MI | ICD-10: I21, I210, I211, I212, I213, I214, I219, I22, I220, I221, I228, I229, I25.2  and  M6551, M6552, M6561, M6563, M6562, M6564, M6553, M6554, M6565, M6566, M6567, M6571, M6572 |  |
| Stroke | ICD-10: I63, I64 | Hospitalized patients |
| Secondary endpoint | All-cause death, myocardial infarction, and stroke individually |  |

ICD, international classification of diseases; EDI, electronic data interchange; MI, myocardial infarction; PAD, peripheral artery disease; ESRD, end-stage renal disease; PCI, percutaneous coronary intervention; CABG, coronary artery bypass graft; STEMI, ST-elevation myocardial infarction; NSTEMI, non-ST elevation myocardial infarction

**Supplementary Table S3.** Baseline characteristics of the matched population using inverse probability of treatment weighting

| Statin group | Inverse probability of treatment weighting | | |
| --- | --- | --- | --- |
|  | Moderate Statin + Ezetimibe | High  Statin | Standardized Difference |
| **Participants** | 10,992.6 (100.0) | 120,129.0 (100.0) |  |
| **Male sex** | 8,179.0 (74.4) | 89,023.5 (74.1) | 0.01 |
| **Age groups** |  |  | 0.04 |
| ≤40s | 1,369.9 (12.5) | 15,231.6 (12.7) |  |
| 50s | 2830.9 (25.8) | 29,656.3 (24.7) |  |
| 60s | 3,039.7 (27.7) | 32,953.9 (27.4) |  |
| 70s | 2,509.9 (22.8) | 28,418.9 (23.7) |  |
| ≥80s | 1,242.1 (11.3) | 13,868.3 (11.5) |  |
| **Comorbidity and revascularization** | | | |
| Diabetes mellitus | 3,208.0 (29.2) | 35,536.0 (29.6) | -0.01 |
| Hypertension | 9,376.9 (85.3) | 100,799.0 (83.9) | 0.04 |
| Old MI | 6,169.2 (56.1) | 64,462.2 (53.7) | 0.05 |
| Heart failure | 1,914.8 (17.4) | 22,440.3 (18.7) | -0.03 |
| PAD | 631.2 (5.7) | 7,145.8 (6.0) | -0.01 |
| ESRD | 325.3 (3.0) | 3,255.5 (2.7) | 0.02 |
| Prior PCI | 920.9 (8.4) | 9,935.1 (8.3) | 0.01 |
| Prior CABG | 928.8 (8.5) | 10,051.4 (8.4) | 0.01 |
| **Clinical presentation at index procedure** | | | |
|  | 10,120.9 (92.1) | 108,882.6 (90.6) |  |
| STEMI | 2,651.1 (24.1) | 28,612.7 (23.8) | 0.01 |
| NSTEMI | 4,573.5 (41.6) | 46,814.2 (39.0) | 0.05 |
| Unstable angina | 2,896.3 (26.4) | 33,455.7 (27.9) | -0.03 |
| **Potent P2Y12 inhibitor** | 2,176.7 (19.8) | 22,561.2 (18.8) | 0.03 |
| **Tertiary general hospital** | 5,481.8 (49.9) | 66,340.5 (55.2) | -0.11 |
| **Years of PCI** |  |  | 0.04 |
| 2013 | 1,019.9 (9.3) | 10,783.1 (9.0) |  |
| 2014 | 1,352.2 (12.3) | 14,048.6 (11.7) |  |
| 2015 | 1,588.1 (14.5) | 15,431.9 (12.9) |  |
| 2016 | 1,546.4 (14.1) | 17,287.9 (14.4) |  |
| 2017 | 1,712.9 (15.6) | 19,206.1 (16.0) |  |
| 2018 | 1,816.5 (16.5) | 20,967.2 (17.5) |  |
| 2019 | 1,956.6 (17.8) | 22,404.2 (18.7) |  |

Data are number (%) or mean ± standard deviation (SD).

MI, myocardial infarction; PAD, peripheral artery disease; ESRD, end-stage renal disease; PCI, percutaneous coronary intervention; CABG, coronary artery bypass graft; STEMI, ST-elevation myocardial infarction; NSTEMI, non-ST elevation myocardial infarction

**Supplementary Table S4.** Risk of clinical outcomes and description of statin adherence in the matched population using inverse probability of treatment weighting

|  | **IPTW-adjusted** | |
| --- | --- | --- |
|  | **Hazard ratio** | ***P* value** |
| **Primary endpoint - Composite of all-cause death, myocardial infarction, and stroke** | 0.91 (0.86-0.95) | 0.000 |
| All-cause death | 0.86 (0.80-0.93) | 0.000 |
| Myocardial infarction | 1.00 (0.91-1.09) | 0.924 |
| Stroke | 0.93 (0.85-1.01) | 0.101 |

Data are number (%).

Hazard ratio for moderate statin + ezetimibe group was derived from Cox proportional analysis.

IPTW, inverse probability of treatment weighting.

**Supplementary Figure S1.** Propensity score distribution before propensity score matching between moderate-intensity statin with ezetimibe therapy and high-intensity statin monotherapy

| **Moderate Statin + Ezetimibe** | **High Statin** |
| --- | --- |
| 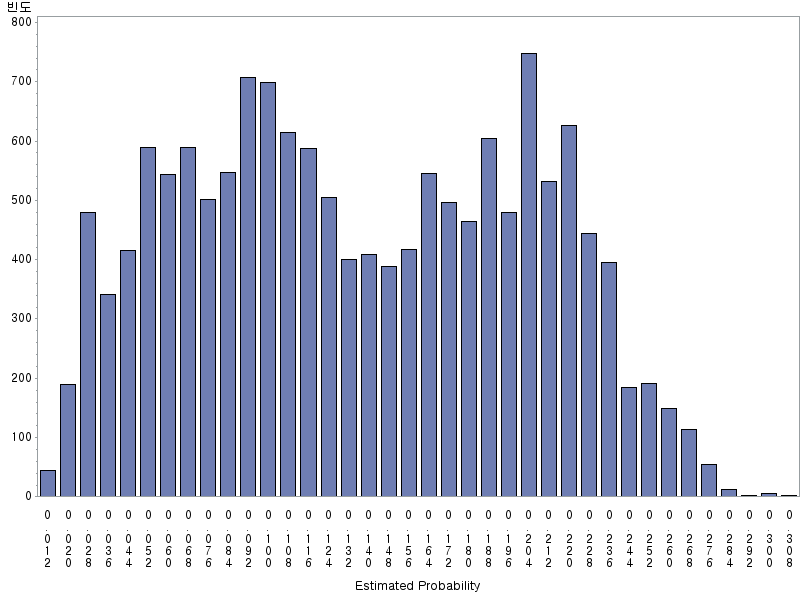 | 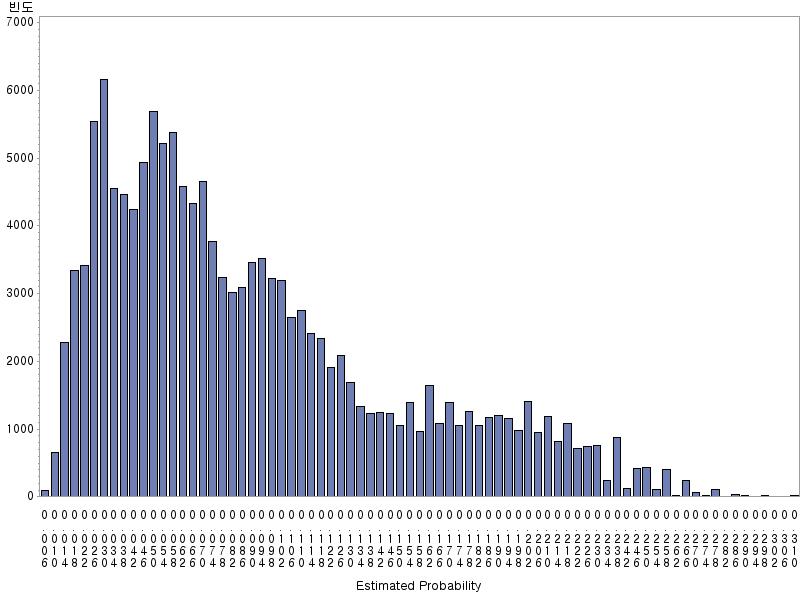 |

The propensity score is use to balance the distribution of baseline covariates between two groups.
